# Testing the impact of local alcohol licencing policies on reported crime rates in England

**DOI:** 10.1136/jech-2016-207753

**Published:** 2016-08-11

**Authors:** F De Vocht, J Heron, R Campbell, M Egan, J D Mooney, C Angus, A Brennan, M Hickman

**Affiliations:** 1 NIHR School for Public Health Research (SPHR), Bristol, UK; 2 School of Social and Community Medicine, University of Bristol, Bristol, UK; 3 Department of Health Services Research and Policy, London School of Hygiene and Tropical Medicine, London, UK; 4 Health Sciences and Well-being Research Group, University of Sunderland, Sunderland, UK; 5 ScHARR, School of Health and Related Research, University of Sheffield, Sheffield, UK

**Keywords:** ALCOHOL, PUBLIC HEALTH, PUBLIC HEALTH POLICY, LONGITUDINAL STUDIES

## Abstract

**Background:**

Excessive alcohol use contributes to public nuisance, antisocial behaviour, and domestic, interpersonal and sexual violence. We test whether licencing policies aimed at restricting its spatial and/or temporal availability, including cumulative impact zones, are associated with reductions in alcohol-related crime.

**Methods:**

Reported crimes at English lower tier local authority (LTLA) level were used to calculate the rates of reported crimes including alcohol-attributable rates of sexual offences and violence against a person, and public order offences. Financial fraud was included as a control crime not directly associated with alcohol abuse. Each area was classified as to its cumulative licensing policy intensity for 2009–2015 and categorised as ‘passive’, low, medium or high. Crime rates adjusted for area deprivation, outlet density, alcohol-related hospital admissions and population size at baseline were analysed using hierarchical (log-rate) growth modelling.

**Results:**

284 of 326 LTLAs could be linked and had complete data. From 2009 to 2013 alcohol-related violent and sexual crimes and public order offences rates declined faster in areas with more ‘intense’ policies (about 1.2, 0.10 and 1.7 per 1000 people compared with 0.6, 0.01 and 1.0 per 1000 people in ‘passive’ areas, respectively). Post-2013, the recorded rates increased again. No trends were observed for financial fraud.

**Conclusions:**

Local areas in England with more intense alcohol licensing policies had a stronger decline in rates of violent crimes, sexual crimes and public order offences in the period up to 2013 of the order of 4–6% greater compared with areas where these policies were not in place, but not thereafter.

## Introduction

Excessive alcohol consumption is known to have not only a negative effect on the health of the individual, but also has wider negative societal impacts such as public nuisance, antisocial behaviour and violence including domestic, interpersonal and sexual.^1–7^

In England and Wales, guidance issued in 2005^8^ extended the 2003 Licensing Act^9^ around the four statutory licensing objectives: (1) the prevention of crime and disorder, (2) maintain public safety, (3) the prevention of public nuisance and (4) the protection of children from harm. This gives local authorities discretionary powers to develop cumulative impact policies, in which, in specific designated areas (cumulative impact zones (CIZ)), the burden of proof during licensing decisions is reversed in that applications for new licences will be refused unless the applicant can demonstrate they will avoid compromising the licensing objectives. These restrictions can take different forms and their implementation varies by local areas,^10^ ^11^ although they all operate under the same policy framework. Two main policy strategies that have been advanced to address alcohol-related harms are modifying the price of alcohol, to make it less affordable, or restricting its spatial and/or temporal availability.^12^ Policies to increase the minimum unit price have not (yet) been implemented.^13^

Public health is not currently included in the statutory licensing objectives. Indeed, the guidance^8^ specifically states that public health cannot be the primary consideration for a licensing decision for individual premises, although it can be used in support of a decision, and can be used to support the case for a cumulative impact policy. Nonetheless, we have shown that the intensity of local licensing policies and enforcement, defined as willingness to implement cumulative impact policies and refusal of licence applications, was associated with a stronger reduction in alcohol-related hospital admissions over time.^14^

Given that the licensing objectives do include the prevention of crime and disorder, maintain public safety and the prevention of public nuisance specifically, a direct effect on alcohol-related crime rates would similarly be expected, but no studies of the effect of policies themselves on crime outcomes—other than through specific mediating factors such as outlet density^15^ ^16^—have been conducted. In this paper, we test whether local licensing policies are associated with reduction in alcohol-related crime.

## Materials and methods

### Data

Quarterly data of reported crimes at lower tier local authority (LTLA) level for England (ie, 55 unitary authorities, City of London and Isles of Scilly, 201 non-metropolitan districts, 36 metropolitan boroughs and 32 London boroughs) were obtained from the UK Office of National Statistics (ONS)^17^ up to the year 2015. Alcohol-related sexual offences and alcohol-related violence against a person (with or without injury) have been calculated by multiplying the reported counts by their alcohol-attributable fraction (AAF). Based on the Local Alcohol Profiles for England (LAPE; http://www.lape.org.uk/), AAFs of 13% and 37% are used to calculate alcohol-related sexual offences and alcohol-related violence against a person, respectively^18^ (ie, 13% of all sexual offences and 37% of all violent offences are assumed to be directly attributable to alcohol consumption). The resulting offence counts were then divided by the corresponding LTLA population sizes for the corresponding year obtained from the UK ONS^19^ to obtain the rates per 1000 people.

We similarly obtained reported counts of public order offences, shoplifting, possession of a weapon, drug offences and domestic burglaries and calculated the annual area rates for each LTLA. Although for the vast majority of crimes these are not attributable to alcohol use, they do correlate (cluster) with alcohol-related sexual and violent offences at area level. We did not multiply these by an AAF since these were not specifically provided in.^18^

‘Financial fraud’ is a crime we classified, a priori, as not being caused by alcohol consumption, and using the same methodology as above, we generated area-level annual rates for these as well as for this ‘control crime’.

These data were further linked at LTLA level to 2009/2010 deprivation data (measured using the Index of Multiple Deprivation) also obtained from the ONS,^20^ and to alcohol outlet density which was obtained from two commercial market research companies who maintain national databases of licenced and trading outlets selling alcohol. Outlet density was calculated as the number of outlets within 1 km of the address-weighted centroid of each postcode: subsequently for each lower super output area (LSOA) the average of the outlet counts within the constituent postcodes, weighted by the corresponding census population of each postcode, was calculated, and these were averaged across all constituent LSOAs to obtain a measure of outlet density for each LTLA. Conceptually this measure captures the average number of outlets within walking distance of an individual within the LTLA.

LTLAs were coded for alcohol policy intensity based on a metric described and used in a previous publication.^14^ In short, data on the presence of CIZ and on successful challenges of licences for new premises in LTLAs were extracted from the 2007/2008 and 2011/2012 alcohol licensing data collected in the Home Office's ‘Alcohol and Late Night Refreshment Licensing England and Wales data’.^21^ Both variables were recoded as present (1) or absent (0) and for each year added together to derive a three-level score that we interpreted as an indication of how active an area's alcohol policy was. We developed a cumulative policy intensity index, which was derived by adding up annual scores for each year and subsequently dividing them in four categories (passive, low, medium and high) based on quartiles of the distribution. This metric was derived to account for the cumulative effect of a policy over time.

There are a total of 326 LTLAs, or districts, in England in 2014, but we were only able to link data for 312 areas; either because of missing data or because of boundary changes during the analysis period. In addition, initial sensitivity analyses identified three areas (City of London, City of Westminster and Isles of Scilly) with consistently high residuals in the statistical models (using an a priori cut-off ±0.5 in multiple years), and these outliers were removed prior to the main analyses. We only included areas with complete data for the years 2009–2015 and excluded areas with <24 data points (ie, <6 years of data), resulting in a final data set of 284 local areas for which we had all data.

### Statistical analyses

The data were analysed statistically using growth curve analysis in R statistical software.^22^ Crime rates were log-transformed and associated to a set of explanatory variables; that is, a log-rate model.^23^ Variability between LTLAs at baseline and individual LTLA time trends was modelled by means of hierarchical random intercept–random slopes mixed-effects models with quarter (eg, January–March to October–December) included as a covariate to account for seasonal trends. When there was evidence of temporal non-linearity quadratic and cubic fits were also explored. Model fit was evaluated based on magnitude and patterns in model residuals, evaluation of Gaussian assumptions of parameter estimates and Bayesian Information Criteria (BIC), since all models were considered equally probably a priori.

## Results

Of the 284 areas with complete covariate data for 2009–2014, 171 (60%) had some form of active policy in place as characterised by our index (ie, having a CIZ in the area, having rejected new alcohol licence applications, or both) in 2009 (table 1). With respect to the covariate distribution across the four levels of our alcohol policy intensity index, areas with more active policies had at baseline, on average, higher levels of area deprivation, density of alcohol outlets, alcohol-related hospital admissions and population size.

**Table 1:** Demographics

Variable		N/mean/range	Per cent
Total number of local authorities		326	100
Local authority in analyses		284	87
Years		2009–2014	
Number of measurements per local authority		24	
Cumulative licensing policy index	Passive	113	40
	Low	41	14
	Medium	83	29
	High	47	17
Outlet density	Passive	22.2	p<0.001
	Low	23.5	
	Medium	27.8	
	High	48.6	
Normalised IMD score*	Passive	0.17	p<0.001
	Low	0.19	
	Medium	0.20	
	High	0.25	
Population size	Passive	128 404	p<0.001
	Low	157 152	
	Medium	179 676	
	High	252 575	
Alcohol-related hospital admissions at baseline†	Passive	150	p<0.001
	Low	151	
	Medium	158	
	High	163	

*Higher is more deprived.

†Age-standardised per 100 000 population.

IMD, Index of Multiple Deprivation.

Tables 2–4 show three versions of our statistical models each, describing increasing complexity, for alcohol-related violent crime rates, alcohol-related sexual crime rates and public order offences, respectively. Here we will describe the results of the most complex model, since here confounder adjustment is most comprehensive. Observed temporal trends were best described as quadratic functions.

**Table 2:** Growth models. 2009–2014 alcohol-related recorded violent crime rate (per 1000 people)

Parameter	Unadjusted (SE)		Adjusted 1 (SE)		Adjusted 2 (SE)	
	Linear trend	Quadratic trend	Linear trend	Quadratic trend	Linear trend	Quadratic trend
Baseline (year 2009)	1.592 (0.046)		1.314 (0.077)		1.368 (0.117)	
Trend (2009–2014)	−0.171 (0.009)	0.015 (0.001)	−0.171 (0.009)	0.015 (0.001)	−0.177 (0.025)	0.015 (0.002)
	p<0.001	p<0.001	p<0.001	p<0.001	p<0.001	p<0.001
Effect on intercept						
No policy	Ref		Ref		Ref	
Low policy	**−0.195 (0.089)**		**−0.229 (0.079)**		**−0.208 (0.079)**	
Medium	**0.147 (0.071)**		0.074 (0.063)		0.114 (0.064)	
High policy	**0.557 (0.085)**		**0.302 (0.079)**		**0.407 (0.085)**	
	p<0.001		p<0.001		p<0.001	
Deprivation at baseline			3.031 (0.275)		4.375 (0.461)	
			p<0.001		p<0.001	
Population at baseline			−0.042 (0.016)		−0.171 (0.027)	
			p=0.010		p=0.035	
Outlet density at baseline			0.004 (0.001)		0.002 (0.001)	
			p<0.001		p=0.082	
Alcohol-related hospital admissions at baseline			−0.002 (0.001)		−0.002 (0.001)	
			p=0.001		p=0.010	
Effects on slope						
Low policy	**0.103 (0.017)**	**−0.009 (0.001)**	**0.103 (0.015)**	**−0.009 (0.001)**	**0.097 (0.017)**	**−0.008 (0.001)**
Medium	**0.032 (0.013)**	**−0.004 (0.001)**	**0.032 (0.013)**	**−0.004 (0.001)**	−0.020 (0.014)	**−0.003 (0.001)**
High policy	**−0.047 (0.016)**	**0.004 (0.001)**	**−0.047 (0.016)**	**0.004 (0.001)**	**−0.078 (0.018)**	**0.006 (0.002)**
	p<0.001	p<0.001	p<0.001	p<0.001	p<0.001	p<0.001
Deprivation					−0.721 (0.098)	0.080 (0.008)
					p<0.001	p<0.001
Population at baseline					0.050 (0.006)	−0.005 (0.000)
					p=0.401	p=0.897
Outlet density at baseline					0.001 (0.000)	−0.000 (0.000)
					p<0.001	p<0.001
Alcohol-related hospital admissions at baseline					0.000 (0.000)	−0.000 (0.000)
					p=0.094	p=0.025
**Variance**	**Estimates**	**Explained**†	**Estimates**	**Explained**†	**Estimates**	**Explained**†
Intercept	0.209	13%	0.158	34%	0.157	34%
Slope	0.003	1%	0.003	1%	0.003	2%
Residual	0.010	1%	0.010	1%	0.010	3%
R^2^	94.6%		94.6%		94.7%	

Bold indicates p<0.05.

All models also adjusted for quarter (ie, 3-month period).

†Explained variance relative to model without explanatory variables (ie, only time trends as fixed effects).

**Table 3:** Growth models. 2009–2014 alcohol-related recorded sex crime rate (per 1000 people)

Parameter	Unadjusted (SE)		Adjusted 1 (SE)		Adjusted 2 (SE)	
	Linear trend	Quadratic trend	Linear trend	Quadratic trend	Linear trend	Quadratic trend
Baseline (year 2009)	−2.136 (0.051)		−2.476 (0.074)		−1.881 (0.136)	
Trend (2009–2014)	−0.144 (0.013)	0.020 (0.001)	−0.144 (0.013)	0.020 (0.001)	−0.388 (0.037)	0.043 (0.003)
	p<0.001	p<0.001	p<0.001	p<0.001	p<0.001	p<0.001
Effect on intercept						
No policy	Ref		Ref		Ref	
Low policy	−0.185 (0.100)		**−0.218 (0.093)**		−0.167 (0.092)	
Medium	0.116 (0.079)		0.043 (0.074)		**0.149 (0.075)**	
High policy	0.481 (0.095)		**0.245 (0.091)**		**0.488 (0.099)**	
	p<0.001		p<0.001		p<0.001	
Deprivation at baseline			2.361 (0.240)		3.074 (0.535)	
			p<0.001		p<0.001	
Population at baseline			−0.013 (0.014)		−0.204 (0.032)	
			p=0.373		p<0.001	
Outlet density at baseline			0.003 (0.001)		0.002 (0.001)	
			p<0.001		p=0.104	
Alcohol-related hospital admissions at baseline			−0.001 (0.000)		−0.004 (0.001)	
			p=0.101		p<0.001	
Effects on slope						
Low policy	0.095 (0.025)	−0.008 (0.025)	**0.095 (0.023)**	**−0.008 (0.002)**	**0.078 (0.025)**	**−0.007 (0.002)**
Medium	0.034 (0.020)	−0.004 (0.002)	0.034 (0.020)	**−0.004 (0.002)**	−0.003 (0.020)	−0.001 (0.002)
High policy	−0.048 (0.024)	0.004 (0.002)	**−0.048 (0.024)**	0.004 (0.002)	**−0.136 (0.027)**	**0.012 (0.002)**
	p<0.001	p<0.001	p<0.001	p<0.001	p<0.001	p<0.001
Deprivation at baseline					−0.319 (0.146)	0.032 (0.014)
					p=0.029	p=0.019
Population at baseline					0.062 (0.087)	−0.005 (0.001)
					p<0.001	p<0.001
Outlet density at baseline					0.001 (0.000)	−0.000 (0.000)
					p=0.060	p=0.011
Alcohol-related hospital admissions at baseline					0.001 (0.000)	−0.000 (0.000)
					p<0.001	p<0.001
**Variance**	**Estimates**	**Explained†**	**Estimates**	**Explained†**	**Estimates**	**Explained†**
Intercept	0.218	9%	0.178	25%	0.172	28%
Slope	0.003	0.3%	0.003	0.3%	0.003	5%
Residual	0.028	0.4%	0.028	0.4%	0.028	1.5%
R^2^	84.1%		84.1%		84.3%	

Bold indicates p<0.05.

All models also adjusted for quarter (ie, 3-month period).

†Explained variance relative to model without explanatory variables (ie, only time trends as fixed effects).

**Table 4:** Growth models. 2009–2014 (alcohol-related) public order offences (per 1000 people)

Parameter	Unadjusted (SE)		Adjusted 1 (SE)		Adjusted 2 (SE)	
	Linear trend	Quadratic trend	Linear trend	Quadratic trend	Linear trend	Quadratic trend
Baseline (year 2009)	1.290 (0.062)		1.010 (0.112)		1.143 (0.172)	
Trend (2009–2014)	−0.227 (0.013)	0.013 (0.001)	−0.227 (0.013)	0.013 (0.001)	−0.310 (0.037)	0.023 (0.003)
	p<0.001	p<0.001	p<0.001	p<0.001	p<0.001	p<0.001
Effect on intercept						
No policy	Ref		Ref		Ref	
Low policy	−0.060 (0.120)		−0.104 (0.118)		−0.068 (0.116)	
Medium	0.141 (0.096)		0.045 (0.094)		0.111 (0.094)	
High policy	0.678 (0.115)		**0.338 (0.117)**		**0.495 (0.124)**	
	p<0.001		p=0.010		p<0.001	
Deprivation at baseline			2.536 (0.396)		4.233 (0.676)	
			p<0.001		p<0.001	
Population at baseline			0.012 (0.023)		−0.189 (0.040)	
			p=0.615		p<0.001	
Outlet density at baseline			0.006 (0.001)		0.005 (0.002)	
			p<0.001		p=0.004	
Alcohol-related hospital admissions at baseline			−0.002 (0.001)		−0.003 (0.001)	
			p=0.11		p=0.027	
Effects on slope						
Low policy	0.069 (0.025)	−0.006 (0.002)	**0.069 (0.025)**	**−0.005 (0.002)**	**0.063 (0.025)**	**−0.005 (0.002)**
Medium	0.069 (0.020)	−0.007 (0.002)	**0.069 (0.020)**	**−0.007 (0.002)**	**0.055 (0.020)**	**−0.007 (0.002)**
High policy	−0.058 (0.024)	0.006 (0.002)	**−0.058 (0.024)**	**0.006 (0.002)**	**−0.080 (0.027)**	**0.006 (0.002)**
	p<0.001	p<0.001	p<0.001	p<0.001	p<0.001	p<0.001
Deprivation at baseline					−1.130 (0.144)	0.135 (0.012)
					p<0.001	p<0.001
Population at baseline					0.067 (0.009)	−0.005 (0.001)
					p<0.001	p<0.001
Outlet density at baseline					0.000 (0.000)	−0.000 (0.000)
					p=0.243	p=0.220
Alcohol-related hospital admissions at baseline					0.001 (0.000)	−0.000 (0.000)
					p<0.001	p<0.001
Variance	**Estimates**	**Explained**†	**Estimates**	**Explained**†	**Estimates**	**Explained**†
Intercept	0.3703	9%	0.352	14%	0.336	18%
Slope	0.006	0%	0.006	0%	0.005	7%
Residual	0.023	3%	0.023	1%	0.022	3%
R^2^	93.2%		93.2%		93.4%	

Bold indicates p<0.05.

All models also adjusted for quarter (ie, 3-month period).

†Explained variance relative to model without explanatory variables (ie, only time trends as fixed effects).

**Figure 1:**
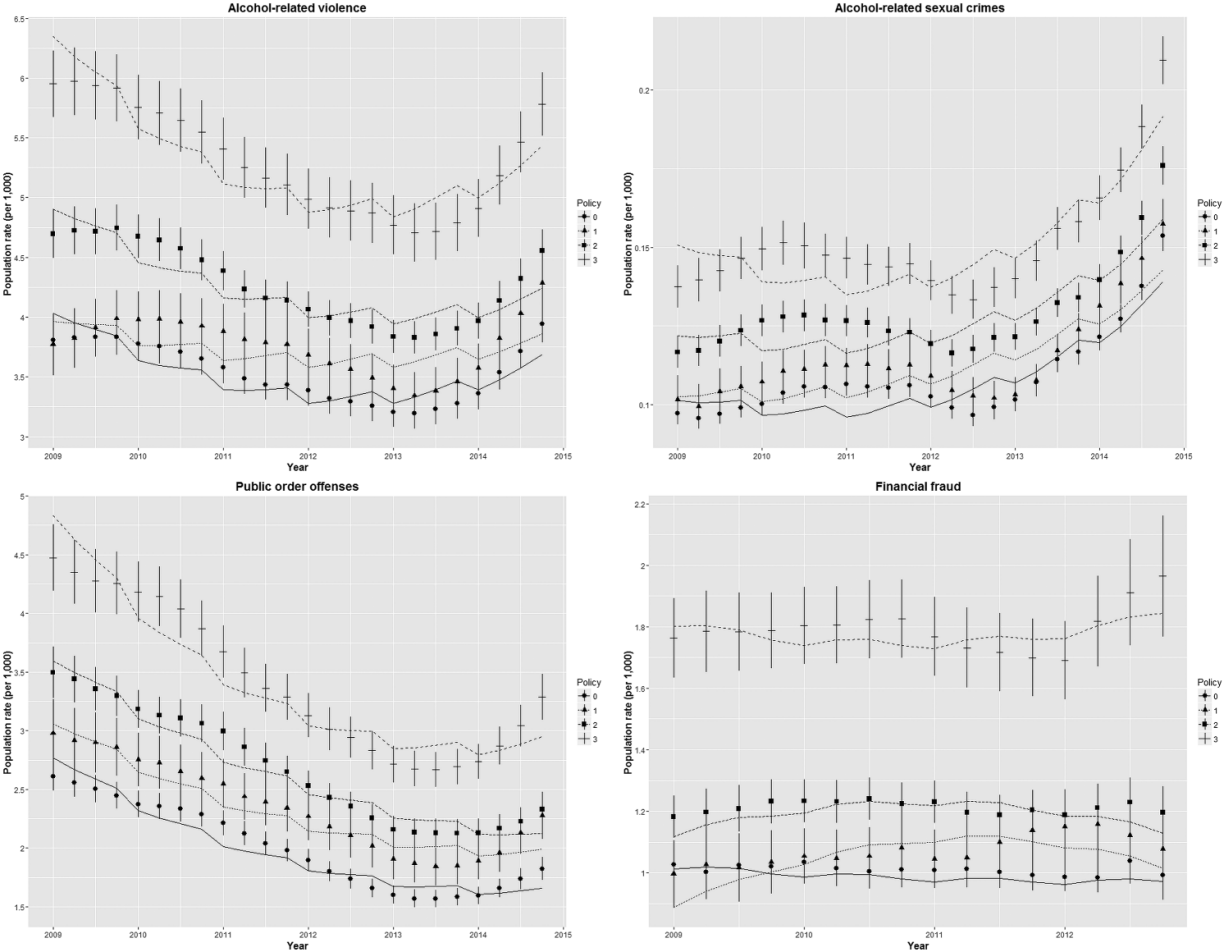
Mean population crime rates (per 1000 people) and SEs of measured data and modelled estimates of adjusted model 2 (lines) for registered alcohol-related crimes and financial fraud. Policy intensity index from ‘passive’ (0) to high (3).

Table 2 describes the results for alcohol-related violent crime rates (with and without personal injury) and indicates that the more intense policies were implemented in the areas with the highest rates. Reported alcohol-related violent crime rates had been reducing until about 2013, but have increased since (figure 1). Their initial decline was statistically significantly different, and steepest, in areas with a more intense alcohol policy compared with the other areas. However, after 2013, the reported rates in all areas increased again, and were faster in areas with more intense alcohol policies, as indicated by the quadratic term. Quantitatively, the effects are relatively moderate and indicate that in the most ‘intense’ areas registered, alcohol-related violent crime rates reduced from about 6.1 per 1000 people in 2009 to 4.9 per 1000 people in 2013 (and back to 5.2 per 1000 people in 2014) compared with a reduction from 3.9/1000 in 2009 to 3.3/1000 in 2013 (and to 3.5/1000 in 2014) in the ‘passive’ areas on average, respectively.

Table 3 and figure 1 similarly describe the results for alcohol-related sexual crimes. The more intense policies were implemented in the areas where baseline sex crime rates were higher. Up until 2013, the rates decreased more in areas with increasing policy intensity (about 0.10 per 1000 people across the period in ‘passive’ areas compared with a reduction from 0.15 to 0.14 per 1000 people in the most ‘intense’ areas). Similar to the above results for violent crimes however, the rate of reported crimes increased most rapidly post-2013 in the areas with the most intense policy.

We found a similar pattern with a stronger reduction in areas with more ‘intense’ alcohol policies for public order offences up to 2013 (table 4; figure 1), but in contrast to alcohol-related violent and sexual crimes, a steep increase post-2013 was absent. More specifically, the registered rates reduced from 2.6 to 1.6 per 1000 people in the passive areas compared with an average decrease from 4.6 to 2.9 per 1000 people in the most ‘intense’ areas.

We compared these results directly with observed trends of ‘financial fraud’, which a priori we classified as not being caused by alcohol consumption and interpreted as a ‘control crime’ (table 5; figure 1). Interestingly, higher rates of financial fraud were present in areas with more intense alcohol policies, which may be associated with the area's level of economic activity, and is comparable to the levels observed for alcohol-related crimes. However, policy-related time trends that were observed for alcohol-related crimes were absent for financial fraud.

**Table 5:** Growth models. 2009–2012 recorded financial fraud rates (per 1000 people). A priori control

Parameter	Financial fraud (SE)	
	Linear trend	Quadratic trend
Baseline (year 2009)	0.502 (0.277)	
Trend (2009–2012)*	−0.200 (0.115)	0.031 (0.014)
	p=0.399	p=0.171
Effect on intercept		
No policy	Ref	
Low policy	**−0.860 (0.186)**	
Medium	−0.172 (0.151)	
High policy	**0.750 (0.200)**	
	p<0.001	
Deprivation	6.409 (1.085)	
	p<0.001	
Population at baseline	−0.598 (0.065)	
	p<0.001	
Outlet density at baseline	0.009 (0.003)	
	p<0.001	
Alcohol-related hospital admissions at baseline	−0.008 (0.002)	
	p<0.001	
Effects on slope		
Low policy	**0.422 (0.077)**	**−0.047 (0.009)**
Medium	**0.167 (0.063)**	**−0.020 (0.008)**
High policy	**−0.187 (0.083)**	**0.020 (0.010**)
	p<0.001	p<0.001
Deprivation at baseline	−2.514 (0.449)	0.074 (0.045)
	p=0.044	p<0.001
Population at baseline	0.291 (0.027)	−0.033 (0.003)
	p<0.001	p<0.001
Outlet density at baseline	−0.002 (0.001)	0.000 (0.000)
	p=0.104	p=0.028
Alcohol-related hospital admissions at baseline	0.002 (0.001)	−0.000 (0.000)
	p=0.019	p=0.001
**Variance**	**Estimates**	**Explained‡**
Intercept	0.501	17%
Slope	0.019	7%
Residual	0.058	4%
	85.3%	

*Post-2012 data not included because reporting changed to national level in 2013.

In addition to the ‘control crime’, we similarly modelled temporal trends in rates of domestic burglaries, shoplifting, possession of weapons and drug offences; offences which, although not directly caused by alcohol consumption, are a likely cluster in similar areas. We observed similar patterns up to 2012, but did not, or only to a very limited extent, observed post-2013 increases in rates (see online supplementary material table S1).

10.1136/jech-2016-207753.supp1 Supplementary material

**Figure 2:**
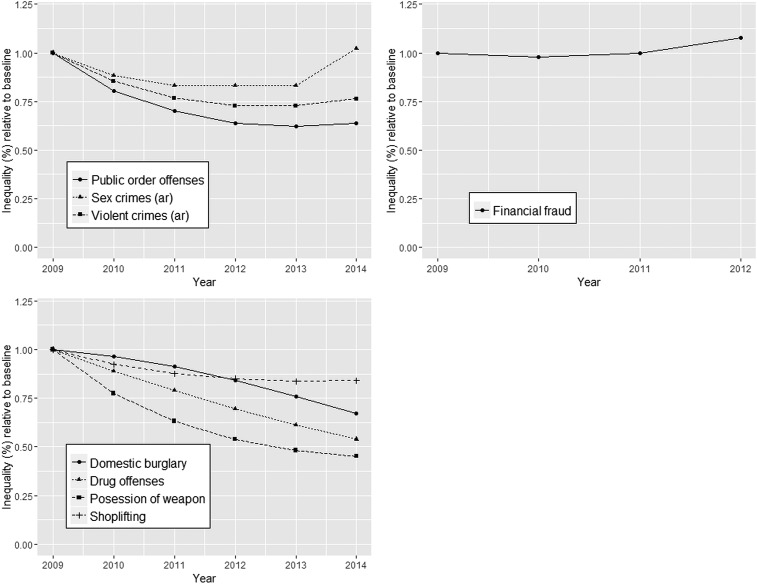
‘Inequality’ is defined as the ratio of annual relative range of local area average crime rates between strata of ‘alcohol policy intensity’ relative to baseline year 2009.

Since the observed non-linear trends are difficult to assess and compare, figure 2 shows the average difference between the areas with the most intense licensing policy compared with the areas with no, or passive, licensing policies with respect to crime rates over time (eg, ‘inequality’ expressed as a ratio relative to baseline year 2009) for all crimes. As shown, inequality decreased (ie, ratio <1) for all alcohol-related crimes as well as the alcohol-correlated crimes during the period up to about 2013, while this is not the case for financial fraud. Note that, as shown in figure 1, this is because crime in highest intensity areas reduces faster than in passive areas; not because crime rates in passive areas increase faster. Post-2012/2013, ‘inequality’ increased again, especially for alcohol-related sexual crimes and to a lesser extent for violent crimes, but stabilised or reduced slower for public order offences, domestic burglary, drug offences, possession of weapons and shoplifting.

## Discussion

These analyses aimed to investigate whether a higher alcohol licensing policy intensity, as characterised by the absence of a CIZ and whether new premises licences had been rejected, was associated with alcohol-related violent crimes, alcohol-related sex crimes and public order offences in a similar way as previously observed for alcohol-related hospital submissions.^14^ Observed temporal trends were not linear across the time period covered, complicating the interpretation of the results in relation to the impact of alcohol licensing policies. Nonetheless, we found stronger reductions in registered alcohol-related violent crimes, alcohol-related sexual crimes and public order offences in areas with more intense alcohol policies and enforcement in the order of an additional 4–6% decrease compared with what would be expected had no policies been in place. Post-2013 however, the rates started to increase again. With respect to absolute differences however, there is evidence of substantial under-reporting of crimes with the ratio of actual (estimated) to reported crime being different for different classes of crimes and ranging from 1:1 (for homicides) to over 1:10 for sexual crimes and shoplifting, making interpretation of absolute effects difficult.^24^ Our study does not allow us to draw any firm conclusions about causality, but we do observe that roughly comparable patterns are observed for crimes we would expect to cluster in the same areas as alcohol-related crimes, while no trends are observed for financial fraud, which as a priori we defined as a ‘control crime’.

To calculate the alcohol-related violent and sex crime rates, we used the methodology employed by the LAPE,^18^ which used AAFs to estimate the alcohol-related fraction of total reported crimes. These are fixed fractions derived from available literature, but nonetheless because these are fixed they are not specific enough to look at specific trends in alcohol-related crimes since AAFs do not vary between LTLAs or over time; which are obviously affected by many other things in society. The most obvious example of this is the Metropolitan Police's highly publicised investigation into sex offences (Operation Yewtree) which resulted in improvements in recording as well as greater willingness of victims to report sexual crimes from 2012 onwards rather than an actual increase in incidence.^25^ More specific data are available from the Crime Survey for England and Wales (CSEW) for only a limited number of territorial police forces where whether a crime was alcohol-related was noted in the data for each crime individually, and for these data up to 2014 a continuing downwards trend in numbers of reported violent crimes where the victim believed the offender(s) to be under the influence of alcohol is indicated.^26^ It is important to realise that reported crime rates are a fraction of the actual crime rates in the community, and the ratio between the two is likely to vary between types of crime, local areas and over time,^24^ while recording practices have also changed over time.^27^ ^28^ It is, however, very difficult to disentangle the causes of observed trends. Questions have been raised about the quality of crime recording and compliance with the Code of Practice for official statistics, while alternatively there is some evidence that the increase or decrease in rates may be the result of genuine changes in the volume of crime.^29^ ^30^ It should also be noted that different AAFs are available from other sources, but these similarly do not vary over the time period covered here.^31^ Nonetheless, although these issues complicated the statistical modelling, as shown by figure 1, we managed to capture the overall trends well assuming a quadratic temporal trend.

As confirmed by the statistical models, alcohol licensing policies, or in wider context policies to reduce antisocial behaviour, are not introduced in random areas, but are introduced in those areas where the cumulative impacts are high. We have adjusted for this in both models by using area-level deprivation, population size, alcohol-related hospital admissions and area outlet density as markers of societal impact of alcohol consumption, but residual confounding may still be present.

Previous work indicated that spatial autocorrelation of the alcohol policy intensity index was negligible,^14^ so we did not adjust for this in these models, while temporal autocorrelation was adjusted for through the use of hierarchical growth models. Drinking patterns in the UK on the other hand have been shown to differ geographically and over time,^32^ ^33^ but we did not have the data to take this into account. There is the possibility of ‘spill-over’ effects, in which new premises open in or move to surrounding areas with less restrictive licensing policies (eg, on the border of CIZ), but data of higher spatial granularity would be required to test this hypothesis.

An important strength of this study is that it makes use of routinely collected and publicly available data, which enables independent replication. Moreover, the linkage of area-level longitudinal data permitted evaluation of time trends in addition to standard cross-sectional studies, which is considered to provide better insight into associations between community-level burdens (from alcohol) and impacts on crime.^4^ ^34^ Another strength of our use of longitudinal data in the particular case of recorded crimes is that, despite problems identified in the recording of these data, quality will be relatively stable over a 6-year period in each local area, enabling analyses of the effect of policies over time without the inference problems from which cross-sectional comparisons would suffer. And finally, because we were able to include different crimes, we were able to evaluate whether observed trends were common features of the different policy strata, or instead were related to the actual policies and were not observed in non-related crimes (ie, financial fraud).

Our analyses confirm previous cross-sectional studies indicating correlations between alcohol outlet density, deprivation and crime rates in the UK and elsewhere,^1–3^ ^5^ ^6^ ^35^ but have now also shown a reverse association between crime rates and alcohol licensing policy intensity, similar to what we observed previously for alcohol-related hospital admissions.^14^ Our main interest however, was to assess temporal trends, for which there is much less data available, especially for the UK.^35^ We observed exposure–effect associations showing that areas with more intense licensing policies had higher decline in alcohol-related violent and alcohol-related sex crimes until 2013, which were not observed for financial fraud and confirm findings from other geographical regions.^36^ The fact that we observed similar trends for reported crimes not directly related to alcohol consumption but spatially clustered with alcohol-related problems, indicate that either the alcohol-licensing policies have a wider impact or, and we consider this more likely, since the four national licensing objectives include crime and disorder, but also public safety, public nuisance and child protection,^37^ licensing policies are part of a wider package of policies and interventions which together have a wider impact than problems from alcohol abuse alone.^11^ Indeed, this cumulative burden from various problems on a community is the reason local authorities consider implementing a CIZ in an area, and this will include stricter alcohol licensing policies but also can include restrictions on other licensable activities (eg, betting shops and hot food take-aways with licences to operate until late at night) and an increased presence of the police, for example.^38^ This accords with previous cross-sectional data showing clustering of crime and alcohol outlet density,^39^ and with ONS data indicating that most alcohol-related violent incidents occur in the evening or night and at the weekend.^26^ It further accords with data from the US, in which in a ‘natural experiment’ setting at microspatial level the repeal of a Sunday alcohol-sales ban was associated with subsequent increase in crime incidents.^40^ If the above is true, then we hypothesise two potential pathways through which licensing intensity leads to reduced crime: (1) more intense licensing leads to less alcohol availability, less (binge) drinking and in turn to less alcohol-related crimes (a pathway from environment to behaviour change), and (2) more intense licensing leads to gentrification, leading to compositional changes (ie, different population groups residing in or going to the area) and this in turn results in reduced crime rates (a pathway from environment to compositional change to less crime).

## Conclusions

These analyses indicate that local areas in England with more intense alcohol licensing policies and enforcement had a stronger decline in rates of violent crimes, sexual crimes and public order offences, at least in the period up to 2013, of the order of 4–6% greater compared with areas where these policies were not in place. Post-2012/2013, selected crime rates have started to increase again, but given that various factors interact, interpretation is difficult.

These findings are in line with previous findings for alcohol-related hospital admissions, but with a still moderate, 2–3 times larger effect. This may be expected, given that the licensing policies aim to directly affect these crimes within the licensing objectives. Although these results cannot be interpreted as causal, they further strengthen the argument about measurable population impacts of alcohol licensing policies.

**What is already known about this subject**

- Excessive alcohol consumption is associated wider negative societal impacts such as antisocial behaviour and violence. All local authorities in England operate under the same policy framework aimed at the prevention of crime and disorder, maintain public safety, prevention of public nuisance, and protection of children from harm. Local differences in how best to address these result in variation in local alcohol licensing policies which could result in different impacts on crime rates.

**What this study adds**

- This paper shows that alcohol-licensing policies in local government areas in England can have a measurable impact on reducing acute negative societal impacts of excessive alcohol consumption. It demonstrates that the intensity and effectiveness of the local licencing policies, including the use of cumulative impact zones, was associated with higher reduction in local rates of violent crimes, sexual crimes and public order offences in the period up to 2013; which was not observed for a negative control not associated with alcohol use. After 2013, however, for a variety of reasons, the rates started to increase again.
