# Supplementary material for: Testing the impact of local alcohol licencing policies on reported crime rates in England
Source: J Epidemiol Community Health. 2016 Aug 11;71(2):137–45. doi: 10.1136/jech-2016-207753 (PMC5284476; doi:10.1136/jech-2016-207753)
Supplement: Supplementary material [file jech-2016-207753supp_material.pdf]

Testing the impact of local alcohol licensing policies on reported crime rates in England

De Vocht F[1,2], Heron J[1,2], Campbell R[1,2], Egan M[1,3], Mooney J[4], Angus C[1,5], Brennan A[1,5], Hickman M[1,2].

[1] NIHR School for Public Health Research (SPHR)

[2] School of Social and Community Medicine, University of Bristol, Bristol, UK

[3] Department of Health Services Research and Policy, London School of Hygiene and Tropical Medicine, London, UK

4 Health Sciences and Well-being Research Group, University of Sunderland, Sunderland, UK

[5] ScHARR, School of Health and Related Research, University of Sheffield, Sheffield, UK

Online Supplementary Material

Disclaimer The views expressed are those of the author(s) and not necessarily those of the NHS, the NIHR or the Department of Health.

Funding This work was funded by the National Institute for Health Research School for Public Health Research (NIHR SPHR). NIHR SPHR is a partnership between the Universities of Sheffield, Bristol, Cambridge, Exeter, UCL; The London School for Hygiene and Tropical Medicine; the LiLaC collaboration between the Universities of Liverpool and Lancaster and Fuse; and The Centre for Translational Research in Public Health, a collaboration between Newcastle, Durham, Northumbria, Sunderland and Teesside Universities.

Licence for Publication: The Corresponding Author has the right to grant on behalf of all authors and does grant on behalf of all authors, an exclusive licence (or non exclusive for government employees) on a worldwide basis to the BMJ Publishing Group Ltd to permit this article (if accepted) to be published in JECH and any other BMJPGL products and sublicences such use and exploit all subsidiary rights, as set out in our licence (http://group.bmj.com/products/journals/instructions-for-authors/licence-forms).

Table. Growth models. 2009-2014 *a priori* selected outcomes related to "alcohol crime environments".

Parameter	shoplifting (SE)		Possession of a weapon (SE)		Drug offenses (SE)		Domestic burglary (SE)	
	**Linear trend**	**Quadratic trend**	**Linear trend**	**Quadratic trend**	**Quadratic trend**	**Quadratic trend**	**Linear trend**	**Quadratic trend**
Baseline (year 2009)	-0.786 (0.162)		-0401 (0.152)		0.556 (0.157)		0.982 (0.164)	
	P<0.001		P<0.001		P<0.001		P<0.001	
Trend (2009-2014)	-0.142 (0.027)	0.008 (0.002)	-0.526 (0.045)	0.042 (0.004)	-0.044 (0.035)	0.008 (0.003)	0.002 (0.034)	-0.009 (0.003)
	P<0.001	P<0.001	P<0.001	P<0.001	P=0.246	P=0.010	P=0.872	P=0.002
**Effect on intercept**								
No policy	ref		ref		ref		ref	
Low policy	-0.073 (0.109)		-0.171 (0.103)		-0.165 (0.106)		**-0.245 (0.111)**	
Medium	**0.148 (0.088)**		0.025 (0.083)		0.020 (0.085)		0.115 (0.090)	
High policy	**0.375 (0.117)**		**0.570 (0.110)**		**0.528 (0.113)**		**0.434 (0.119)**	
	P=0.004		P<0.001		P<0.001		P<0.001	
Deprivation at baseline	3.278 (0.638)		5.832 (0.599)		5.739 (0.616)		-4.961 (0.646)	
	P<0.001		P<0.001		P<0.001		P<0.001	
Population at baseline	-0.20 (0.038)		-0.298 (0.036)		-0.171 (0.037)		-0.175 (0.038)	
	P<0.001		P<0.001		P<0.001		P<0.001	
Outlet density at baseline	0.002 (0.002)		0.001 (0.001)		0.010 (0.002)		0.000 (0.002)	
	P=0.285		P=0.715		P<0.001		P=0.799	
Alcohol-related hospital admissions at baseline	-0.000 (0.001)		-0.004 (0.001)		-0.006 (0.001)		-0.002 (0.001)	
	P=0.895		P=0.001		P<0.001		P=0.119	
**Effects on slope**								
No policy	ref	ref	ref	ref	ref	ref	ref	ref
Low policy	**0.062 (0.018)**	**-0.006 (0.002)**	**0.104 (0.030)**	**-0.105 (0.003)**	**0.087 (0.023)**	**-0.008 (0.002)**	**0.095 (0.023)**	**-0.008 (0.002)**
Medium	-0.011 (0.015)	0.001 (0.001)	0.027 (0.024)	-0.003 (0.002)	0.019 (0.019)	-0.002 (0.002)	0.003 (0.019)	-0.000 (0.002)
High policy	**-0.079 (0.020)**	**0.007 (0.002)**	**-0.166 (0.032)**	**0.014 (0.003)**	**-0.094 (0.025)**	**0.008 (0.002)**	**-0.083 (0.025)**	**0.007 (0.002)**
	P<0.001	P<0.001	P<0.001	P<0.001	P<0.001	P<0.001	P<0.001	P<0.001
Deprivation	-0.700 (0.106)	0.069 (0.009)	-0.773 (0.175)	0.080 (0.017)	-1.066 (0.136)	0.092 (0.012)	-0.682 (0.135)	0.054 (0.012)
	P<0.001	P<0.001	P<0.001	P<0.001	P<0.001	P<0.001	P<0.001	P<0.001
Population at baseline	0.071 (0.006)	-0.006 (0.001)	0.112 (0.010)	-0.011 (0.001)	0.076 (0.008)	-0.008 (0.001)	0.118 (0.008)	-0.012 (0.001)
	P<0.001	P<0.001	P<0.001	P<0.001	P<0.001	P<0.001	P<0.001	P<0.001
Outlet density at baseline	0.001 (0.000)	-0.000 (0.000)	0.001 (0.000)	-0.000 (0.000)	-0.001 (0.000)	0.000 (0.000)	0.001 (0.000)	-0.000 (0.000)
	P<0.001	P<0.001	P=0.003	P=0.003	P=0.002	P=0.042	P=0.002	P=0.002
Alcohol-related hospital admissions at baseline	0.000 (0.000)	-0.000 (0.000)	0.001 (0.000)	-0.000 (0.000)	0.002 (0.000)	-0.000 (0.000)	-0.001 (0.000)	0.000 (0.000)
	P=0.049	P=0.797	P<0.001	P<0.001	P<0.001	P<0.001	P<0.001	P<0.001

Variance estimates	estimates	estimates	estimates	estimates
Intercept	0.317	0.194	0.271	0.305
Slope	0.002	0.003	0.004	0.004
covariance	-0.63	-0.63	-0.75	-0.66
Residual	0.013	0.041	0.021	0.021
BIC	-7,264	-127	-4,272	-4,274
Explained variance	95.9%	89.0%	93.6%	94.5%
